# cGAS knockout inhibited endotoxin-induced uveitis in mice

**DOI:** 10.1016/j.gendis.2025.101786

**Published:** 2025-07-29

**Authors:** Yue Guo, Ruiping Gu, Jiaojiao Wei, Chunhui Jiang

**Affiliations:** a Department of Ophthalmology, Eye and ENT Hospital of Fudan University, Shanghai 200031, China; b Shanghai Key Laboratory of Visual Impairment and Restoration, Fudan University, Shanghai 200031, China; c Eye Institute, Eye and ENT Hospital of Fudan University, Shanghai 200031, China; d Key Laboratory of Myopia of State Health Ministry, Shanghai 200031, China; e Department of Ophthalmology, Peking University People’s Hospital, Beijing 100044, China

**Keywords:** cGAS, EIU, Lipopolysaccharide, Retina, STING

## Abstract

Our research focused on the impact of the cyclic guanosine monophosphate-adenosine monophosphate synthase (cGAS)–stimulator of interferon genes (STING) pathway on retinal inflammation and employed an endotoxin-induced uveitis (EIU) model. EIU was provoked in mice through the intravitreal administration of lipopolysaccharide. Transcriptome analysis was performed via bulk RNA sequencing. Cytosolic mitochondrial DNA levels in the retina were quantified via PCR. Western blotting was used to assess the activation of cGAS‒STING signaling at specified times after intravitreal lipopolysaccharide injection. To understand the influence of the cGAS‒STING pathway on inflammatory retinal disorders, Cgas knockout mice were developed. Fundus imaging and fluorescein angiography were conducted to observe vitreous inflammation. Microstructural analysis of the eyes was performed, and histopathological scoring was performed. Retinal leukocytosis assays were used to evaluate retinal inflammation. Analysis of these differentially expressed mRNAs revealed activation of the cGAS‒STING signaling pathway, which was confirmed by western blotting analysis of these proteins. Using Cgas knockout mice, we observed significant inhibition of endotoxin-induced intraocular inflammation, including reduced vitreous inflammation, reduced retinal vascular leakage, decreased leukocyte adhesion, inhibited infiltration and activation of macrophages in the retina, and inhibited microglial activation. These findings suggest that cGAS might be a potential novel therapeutic target for uveitis.

## Introduction

Cyclic guanosine monophosphate-adenosine monophosphate synthase (cGAS) functions as an essential sensor in the cytoplasm for DNA, pivotal in recognizing and modulating the cellular reaction to DNA from both microbes and the host, identified as danger-associated molecular patterns.^1^, ^2^, ^3^, ^4^ When cGAS encounters cytoplasmic double-stranded DNA, it synthesizes 2,3-cyclic guanosine adenosine monophosphate (2,3-cGAMP), which then activates stimulator of interferon genes (STING).^5^ STING is then activated and translocated to intermediate regions between the endoplasmic reticulum and the Golgi apparatus. This activation triggers additional signaling pathways by recruiting and activating TANK-binding kinase 1 (TBK1), which in turn facilitates the recruitment and phosphorylation of interferon regulatory factor-3 (IRF3). Phosphorylated IRF3 dimerizes and translocates to the nucleus, where it drives the transcription of genes responsible for producing various cytokines, interferons, and chemokines.^6^ Consequently, the cGAS‒STING pathway has been recognized as a crucial mediator of inflammation related to infections, cellular stress, and tissue damage.

Extensive research has underscored the role of the cGAS‒STING pathway across a spectrum of diseases, such as infections,^5^ autoimmune disorders,^7^ cancers,^8^ fibrotic conditions,^9^ and neurodegenerative diseases.^10^, ^11^, ^12^ Inhibiting this pathway has been shown to mitigate inflammatory disease progression, suggesting its potential as a therapeutic target for autoimmune and inflammatory conditions.^6^ The presence of the cGAS‒STING pathway has been reported in several ocular diseases, such as ocular surface inflammation,^13^ diabetic retinopathy,^14^ uveal melanoma,^15^ and age-related macular degeneration.^16^ Under stress, mitochondrial DNA (mtDNA) escapes into the cytoplasm via the mitochondrial permeability transition pore, activating the cGAS-STING pathway and exacerbating inflammatory responses in ocular surface injury and retinal degeneration.^13^^,^^16^ Both genetically and pharmacologically targeting cGAS and blocking mtDNA release effectively attenuated inflammation. Our previous research revealed that pathological stimuli could trigger the release of retinal mtDNA into the cytosol, where it acts as a danger-associated signal detected by cGAS.^17^

Uveitis comprises a range of vision-threatening conditions marked by inflammation of the uvea, including the iris, ciliary body, and choroid. Globally, uveitis affects approximately 50 individuals per 100,000 annually, representing 10%–15% of blindness cases in developed nations and 25% in developing regions.^18^, ^19^, ^20^, ^21^ Endotoxin-induced uveitis (EIU) is an animal model of acute uveitis that is induced by administering sublethal doses of exogenous bacterial toxins, such as lipopolysaccharide (LPS). The EIU model faithfully recapitulates key pathological features of human acute uveitis. Both EIU and human disease exhibit elevated levels of proinflammatory cytokines (TNF-α, IL-6, IL-1β, and MCP-1), with IL-6 levels correlating positively with neutrophil infiltration. Pathologically, LPS-induced vascular permeability and cellular exudation in EIU mirror the acute inflammatory phenotype observed clinically. Notably, retinal Müller cell activation, marked by up-regulated glial fibrillary acidic protein (GFAP) and cytokine secretion, is shared across both systems. These parallels establish EIU as a robust platform for mechanistic studies and therapeutic screening in uveitis.^22^ EIU is commonly utilized for studying the pathological mechanisms of ocular inflammation as well as evaluating the effectiveness of anti-inflammatory agents.^23^^,^^24^ In this study, we demonstrated evidence for the activation of the cGAS‒STING pathway in a mouse model of LPS-induced uveitis.^25^ We observed that knockout (KO) of cGAS significantly diminished the ocular inflammatory response following intravitreal LPS injection in mice. These results suggest a novel therapeutic approach targeting the cGAS‒STING pathway for managing uveitis.

## Materials and methods

### Animals

Wild-type C57BL/6J mice were acquired from Spfbiotech, Beijing, China, while Cgas KO C57BL/6J mice were kindly supplied by H. Yang (Fudan University). The 8-week-old mice were maintained on a 12-h/12-h light/dark cycle at temperatures between 22 °C and 24 °C. To induce EIU in mice, 1 μL of LPS solution (125 ng/μL) was injected intravitreally. The animals were anesthetized intraperitoneally with 1.25% tribromoethanol (0.4 mL/20 g), and their pupils were dilated with tropicamide and phenylephrine (Santen Pharmaceuticals, Osaka, Japan). A microinjector (Hamilton, Reno, NV, USA) was used for LPS delivery. The LPS solution (1 μL, 125 ng/μL) was injected posteriorly to the limbus. The control injections used phosphate-buffered saline (PBS; 1 μL; Cat# 10010023; Thermo Scientific, USA). After injection, the mice were returned to standard housing conditions and divided into four groups: WT with PBS (WT NC group), WT with LPS (WT EIU group), Cgas KO with PBS (KO NC group), and Cgas KO with LPS (KO EIU group). All procedures received approval from the Animal Ethics Committee at the EENT Hospital of Fudan University, Shanghai, China, and adhered to the guidelines set by the Association for Research in Vision and Ophthalmology.

### RNA isolation from retinas

Forty-eight hours after injection, retinal RNA was isolated via a total RNA isolation kit (Thermo Fisher Scientific), and its integrity was verified via a 2100 Bioanalyzer (Agilent Technologies, Santa Clara, CA). The RNA libraries were then generated via the VAHTS Universal V6 RNA-seq Library Prep Kit. Sequencing was conducted on an Illumina NovaSeq 6000 platform, yielding 150 bp paired-end reads. The initial fastq data were filtered with fastp to remove low-quality sequences, providing clean reads. These reads were subsequently aligned to the reference genome via HISAT2. Gene expression levels were expressed as fragments per kilobase of exons (FPKM), and gene counts were determined through HTSeq-count. To evaluate reproducibility, principal component analysis was carried out via R (v3.2.0).

### Expression analysis of differentially expressed transcripts, GO enrichment analysis, KEGG pathway enrichment analysis, and TF binding site enrichment analysis

The mRNA expression levels were determined via the FPKM method. Transcripts with a fold change (FC) ≥ 2.0 and *P* < 0.05 were classified as differentially expressed between the EIU and NC groups, as well as between the WT EIU and KO EIU groups. The genomic distribution of the differentially expressed mRNAs was visualized via Circos (http://circos.ca/). Gene Ontology (GO) enrichment analysis, Kyoto Encyclopedia of Genes and Genomes (KEGG) pathway analysis, and transcription factor (TF) binding site enrichment analysis were subsequently conducted to elucidate the potential biological functions of these transcripts. Genes with *P* < 0.05 were deemed significantly enriched in specific terms.

### Isolation of cytosolic supernatant and detection of cytosolic mtDNA

At 24 and 48 h post-intravitreal injection, the anterior eye segments were removed, and the retinas were carefully dissected. Cytosolic mtDNA isolation was performed following a previously described protocol.^17^ The retinas were weighed, minced, and subjected to a mitochondria isolation kit (C3606; Beyotime, Shanghai, China) following the manufacturer’s protocol. The retinas were combined with ten volumes of separation reagent A and homogenized. The supernatant was subsequently centrifuged at 11,000 *g* at 4 °C for 10 min to separate the mitochondrial pellet from the cytosolic fraction. The detection of cytosolic mtDNA was conducted via PCR as previously described.^17^ Quantitative PCR was performed to detect mtDNA via primers for mitochondrial cytochrome c oxidase 1 (mt–Co1) and nuclear DNA with primers for 18S rDNA. The primers used are listed in Table S1.

### Western blotting

Retinas were collected 24 and 48 h after LPS or PBS injection. Whole retina tissue was lysed on ice with RIPA buffer (P0013B; Beyotime). After ultrasonication, the lysates were centrifuged at 12,000 *g* for 10 min, and the supernatant was collected in a 1.5 mL tube to measure the protein concentrations. Then, the proteins were resolved on a 4%–20% Tris–glycine gel and transferred to polyvinylidene difluoride membranes. The membranes were blocked with bovine serum albumin and incubated overnight with primary antibodies, as detailed in Table S2. Secondary antibodies were then applied. Immunoblots were detected and quantified using Pierce Enhanced Chemiluminescence Western Blot Substrate (Thermo Fisher Scientific).

### Fundus photography and fundus fluorescein angiography

Forty-eight hours after LPS or PBS injection, the mice were anesthetized with 1.25% tribromoethanol, and their pupils were dilated with tropicamide and phenylephrine eye drops (Santen Pharmaceuticals). Carbomer ophthalmic gel (Bausch & Lomb, Rochester, New York, USA) was applied to the corneas. Fundus images were obtained via a small-animal fundus imaging system (OPTO-RIS; Optoprobe Science, UK). The grade of vitreous opacity reflects the severity of vitreous inflammation. The severity of vitreous opacity was graded from 0 to 3 via the following scoring system^26^: grade 0 = clear fundus and vitreous; grade 1 = small areas of vitreous haze with some visible fundus details and a good red reflex; grade 2 = moderate vitreous haze with no visible fundus details and a partial red reflex; and grade 3 = no red reflex. For fundus fluorescein angiography, the mice received an intraperitoneal injection of 1.7 mL/kg 2% fluorescein sodium.

### Histopathologic evaluation

Forty-eight hours after LPS or PBS intravitreal injection, eyes were preserved in FAS eyeball fixative solution, subsequently dehydrated, and embedded in paraffin. Retinal tissue sections were sliced vertically through the optic nerve and subjected to hematoxylin-eosin staining. The inflammatory cell counts of the anterior and posterior segments were assessed. The histologic grade of inflammation was scored on a scale from 0 to 3 as outlined in reference^27^: grade 0 = no cell infiltration; grade 1 = mild infiltration (0–75 cells); grade 2 = moderate infiltration (76–150 cells); and grade 3 = severe infiltration with exudates (>150 cells).

### Retinal leukostasis assay

Retinal leukostasis was evaluated 48 h after intravitreal injection. Concanavalin A and fluorescein (FL-1001-25; Vectorlabs) were dissolved in PBS. The mice were euthanized and perfused with prewarmed PBS containing 20 μg/mL concanavalin A through the left ventricle. After perfusion, the eyes were fixed in 4% paraformaldehyde at room temperature for 30 min. After rinsing with PBS, the anterior segments were excised. The retinas were flat-mounted and examined with a confocal laser scanning microscope (Leica STED, 495/515 nm). Leukocytes adhering to retinal blood vessels were quantified with ImageJ software. Each experimental group included six mice.

### Flow cytometry

The mice were euthanized, and their eyes were enucleated to isolate the retinas. Four retinas were pooled in 1 mL of digestion buffer containing Hibernate medium (HBSS, Corning) supplemented with 5% fetal bovine serum, 10 mM HEPES, 0.7 mg/mL calcium chloride, 1.5 mg/mL collagenase A, and 0.1 mg/mL DNase I. The tissue was incubated at 37 °C for 15–20 min with gentle trituration, and the samples were prepared for single-cell sequencing and flow cytometry analysis. After digestion, the resulting cell suspension was filtered through a 70-μm cell strainer (Genesee Scientific) and resuspended in PBS. Mononuclear macrophages were isolated via an Animal Tissue Mononuclear Cell Isolation Solution Kit (Cat# P5390; Beijing Solarbio Science & Technology Co., Ltd., China). The isolated retinal macrophages were blocked with 5% fetal bovine serum for 5 min and then incubated with phycoerythrin-conjugated anti-mouse CD11b (BioLegend Cat# 101207) and fluorescein-conjugated anti-mouse CD45 (BioLegend Cat# 157213) antibodies at 4 °C in the dark for 30 min. For regulatory T cells (Tregs), the cells were incubated with Alexa Fluor® 647-conjugated anti-mouse FOXP3 (BioLegend Cat# 113704), fluorescein-conjugated anti-mouse CD4 (BioLegend Cat# 100405), and phycoerythrin-conjugated anti-mouse CD25 (BioLegend Cat# 113703) antibodies. After staining, the cell suspensions were washed twice with 0.5% bovine serum albumin and centrifuged at 500 *g* for 5 min. The final cell suspensions for flow cytometry analysis were fixed with 4% paraformaldehyde. All flow cytometry analyses were conducted via a BD FACSCalibur, and FlowJo software was used for data analysis.

### Immunofluorescence staining

Following enucleation, the eyes were immediately placed in 4% paraformaldehyde at room temperature for 30 min to fix the tissues. After fixation, the anterior segments and lenses were carefully removed. The eyecup was then subjected to 4–5 radial cuts to facilitate separation of the retina from the eyecup via small forceps, resulting in a retinal flat mount. After three washes with PBS, the retinas were incubated in 0.3% Triton X-100 for 30 min and subsequently blocked with 5% goat serum for 1 h at 37 °C. The retinas were then incubated at 4 °C overnight with the primary antibody (recombinant anti-Iba1 antibody, EPR16588, diluted 1:200, ab178846, Abcam). Next, the retinas were incubated with a secondary antibody (goat anti-rabbit IgG H&L, Alexa Fluor® 488, Abcam) for 60 min and counterstained with 4′,6-diamidino-2-phenylindole (DAPI; Sigma–Aldrich, St. Louis, Missouri, USA) for 15 min. Retinal tissues were then visualized via a laser confocal microscope (Leica Microsystems). ImageJ software was used to analyze the endpoints, branch points, and branch lengths of the microglia.

### Statistical analysis

Statistical analyses were carried out via GraphPad Prism (Dotmatics, Boston, Massachusetts, USA). The Mann–Whitney *U* test was used to compare two groups, whereas one-way ANOVA was applied for comparisons involving three or more groups. The data were shown as mean ± standard deviation, with a *P* value ≤ 0.05 considered statistically significant. For RNA-sequencing data analysis, the differential expression of protein-coding genes was determined via DESeq2, which normalizes raw counts via the base mean method and performs statistical testing with a negative binomial model (adjusted *P* value < 0.05, |log_2_(fold change)| > 1). In studies lacking biological replicates, DESeq was employed as the default workflow. The quantitative PCR data were analyzed via a two-tailed Student’s *t*-test.

## Results

### Analysis and validation of differentially expressed mRNAs

**Figure 1:**
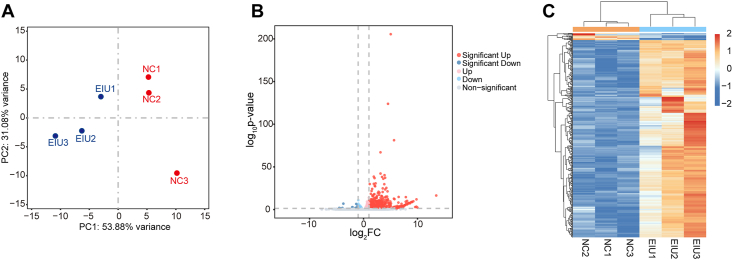
Analysis and confirmation of the differentially expressed mRNAs in the NC and EIU groups. **(A)** Principal component analysis of retinal mRNA expression levels in the NC and EIU groups. The distribution of dots indicates distinct mRNA expression patterns between the normal control and EIU groups. **(B)** Volcano plot of total mRNAs. The red, blue, and gray dots represent up-regulated, down-regulated, and non-significantly altered mRNAs, respectively. The data at infinity are not shown. **(C)** Hierarchical clustering heatmap analysis of differentially expressed mRNAs. The red represents increased expression, and the blue represents decreased expression. NC, normal control; EIU, endotoxin-induced uveitis.

To analyze the mRNA distribution across various samples, principal component analysis was performed, confirming a clear separation between the EIU and normal control (NC; PBS-treated) groups (Fig. 1A) at 48 h post-intravitreal injection. Following the filtering criteria detailed, we identified 438 mRNAs with differential expression, with 423 up-regulated and 15 down-regulated (Table S3). The volcano plot and hierarchical clustering heatmap of these mRNAs are shown in Figure 1B and C.

### GO enrichment analysis, KEGG pathway analysis, TF binding site enrichment analysis, and cytosolic mtDNA release

**Figure 2:**
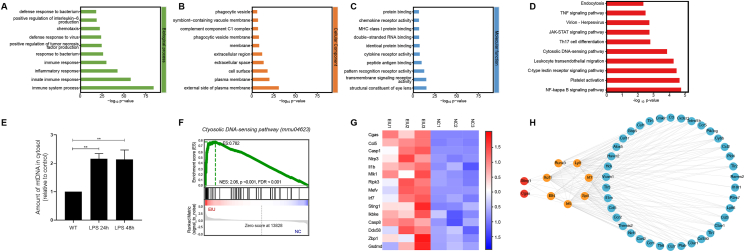
GO enrichment analysis and KEGG pathway analysis of co-expressed mRNA targets in the NC and EIU groups. **(A**–**C)** GO enrichment analysis of co-expressed mRNA targets, covering biological processes, cellular components, and molecular functions. The top 10 GO terms of each domain are shown. **(D)** The top 10 enriched KEGG terms. **(E)** Real-time PCR was used to quantify the number of miDNA copies in the cytoplasm. The released mtDNA was significantly increased at 24 h and 48 h in the EIU groups. ∗∗*P* < 0.01. *n* = 3 biological replicates in each group. **(F)** GSEA snapshots of cytosolic DNA-sensing pathway enrichment analysis. **(G)** Hierarchical clustering heatmap analysis of cytosolic DNA-sensing pathway mRNAs. **(H)** The cGAS-STING-TF-inflammatory network. NC, normal control; EIU, endotoxin-induced uveitis; GSEA, gene set enrichment analysis; GO, gene ontology; KEGG, Kyoto Encyclopedia of Genes and Genomes; cGAS, cyclic guanosine monophosphate-adenosine monophosphate synthase; STING, stimulator of interferon genes; TF, transcription factor.

GO enrichment analysis of the differentially expressed mRNA targets revealed 790 significantly enriched GO terms (Table S4). The top ten enriched GO terms across biological processes, molecular functions, and cellular components are presented in Figure 2A–C. As shown in Table S4, the GO enrichment analysis revealed notable enrichment in immune system process (GO:0002376, *P* < 0.001), innate immune response (GO:0045087, *P* < 0.001), defense response to virus (GO:0051607, *P* < 0.001), activation of the innate immune response (GO:0002218, *P* < 0.001), regulation of the immune response (GO:0050776, *P* < 0.001), positive regulation of type I interferon production (GO:0032481, *P* < 0.001), positive regulation of the defense response to the virus by the host (GO:0002230, *P* < 0.001), and regulation of type I interferon production (GO:0032479, *P* = 0.01). KEGG pathway analysis was further conducted, with the top 10 enriched KEGG terms shown in Figure 2D. The cytosolic DNA-sensing pathway was significantly enriched in the EIU retina (KEGG: mmu04623, *P* = 0.00014). These enrichment terms indicate that the cGAS signaling pathway may be involved in the EIU model of retinal inflammation.

Previous research has demonstrated that pathological conditions can cause the release of mtDNA into the cytoplasm, where it is recognized as exogenous dsDNA by cGAS.^17^ Accordingly, real-time PCR was utilized to quantify mtDNA levels in the retinal cytoplasm. We observed a significant increase in cytosolic mtDNA in the EIU retina (Fig. 2E). Additionally, gene set enrichment analysis (GSEA) revealed substantial enrichment of gene sets associated with the cytosolic DNA-sensing pathway in the EIU retina (Fig. 2F, G). These results suggest that intravitreal LPS administration in the EIU model triggers mtDNA release into the cytoplasm, thereby activating the cytosolic DNA-sensing pathway. This activation, mediated by the cGAS-STING pathway, enhances inflammatory signals and contributes to retinal damage. To further explore how cGAS-STING signaling orchestrates the inflammatory gene expression program in the EIU retina, TF binding site enrichment analysis was performed on the cGAS-STING-related inflammatory response gene set, and the cGAS-STING-TF-inflammatory network was constructed (Fig. 2H). cGAS might regulate retinal inflammation through TFs.

### EIU activates the cGAS-STING signaling pathway

**Figure 3:**
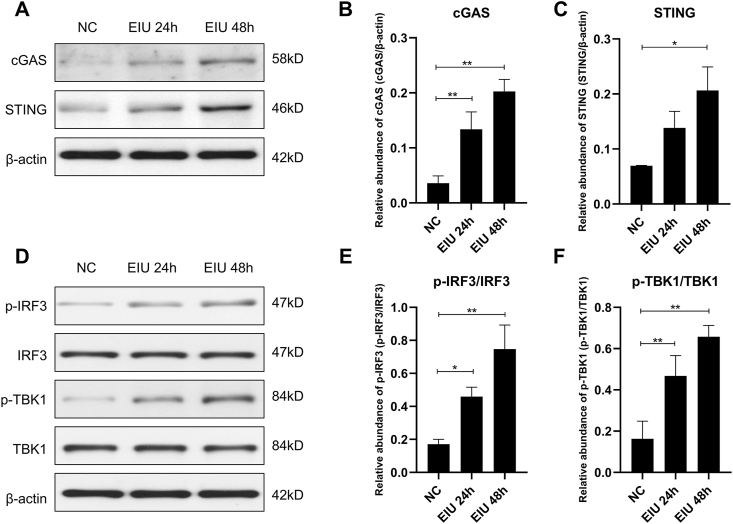
Western blotting analysis of activation of the cGAS/STING pathway **(A–C)** and phosphorylation of IRF3 and TBK1 **(D–F)** in the retina at 24 and 48 h in the EIU groups. ∗*P* < 0.05, ∗∗*P* < 0.01. *n* = 3 biological replicates per group. cGAS, cyclic guanosine monophosphate-adenosine monophosphate synthase; STING, stimulator of interferon genes; IRF3, interferon regulatory factor 3; TBK1, TANK-binding kinase 1; NC, normal control; EIU, endotoxin-induced uveitis.

To assess the activation of the cGAS‒STING pathway in the EIU model, we conducted western blotting to determine protein expression. The expression levels of cGAS and STING were significantly increased at 24 and 48 h after LPS injection (Fig. 3A–C). Additionally, the phosphorylation of TBK1 and IRF3, downstream targets of the cGAS-STING pathway, was notably increased at both time points (Fig. 3D–F).

### Vitreous inflammation and retinal vascular leakage are reduced in Cgas KO mice

**Figure 4:**
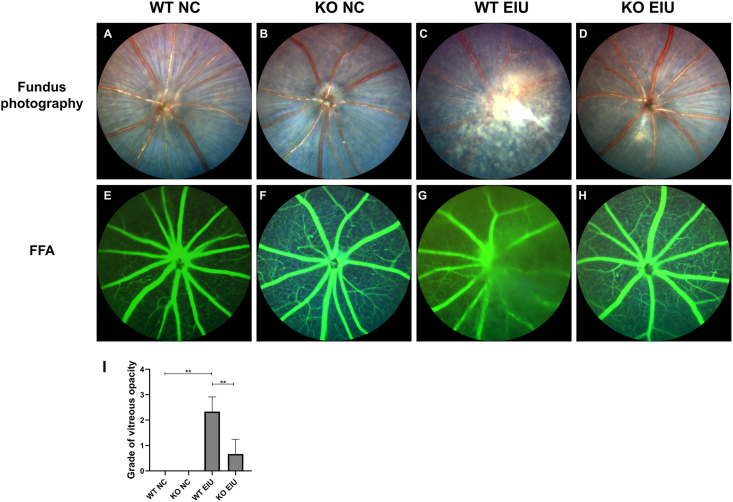
Representative fundus photographs **(A–D)** and FFA **(E–H)** of the retinas in the NC and EIU groups. (A) Fundus photograph from the WT NC group. (B) Fundus photograph from the KO NC group. (C) Fundus photograph from the WT EIU group. (D) Fundus photograph from the KO EIU group. (E) FFA from the WT NC group. (F) FFA from the KO NC group. (G) FFA from the WT EIU group. (H) FFA from the KO EIU group. (I) Grade of the severity of vitreous opacity. ∗∗*P* < 0.01. *n* = 3 biological replicates per group. FFA, fundus fluorescein angiography; WT, wild type; KO, Cgas knockout; NC, normal control; EIU, endotoxin-induced uveitis.

As shown in the fundus images (Fig. 4A–D), the eyes from the KO NC group displayed a normal morphology, similar to those from the WT NC group. Compared with those in the WT EIU group, fundus photographs revealed vitreous opacities and vitreous inflammation in the WT LPS group (Fig. 4C). However, the images of the KO EIU group contained small areas of vitreous haze and reduced vitreous inflammation in the posterior segments (Fig. 4D). The vitreous opacity grades are shown in Figure 4I. The grades ranged from 2 to 3 in the WT EIU group compared with 0–1 in the KO EIU group. The mean vitreous opacity grade was significantly lower in the KO EIU group than in the WT EIU group (WT EIU *vs*. KO EIU: 2.33 ± 0.58 *vs*. 0.67 ± 0.58, *P* = 0.0046; Fig. 4I).

Compared with those in the WT NC group, fluorescein angiography revealed vascular boundary blurring and increased fluorescein leakage in the WT EIU group (Fig. 4E–G). The accumulation of fluorescein in the vitreous chamber was significantly lower in the KO EIU group (Fig. 4H) than in the WT EIU group (Fig. 4G).

### Cgas KO decreased leukocyte adhesion and reduced the number of infiltrating cells

**Figure 5:**
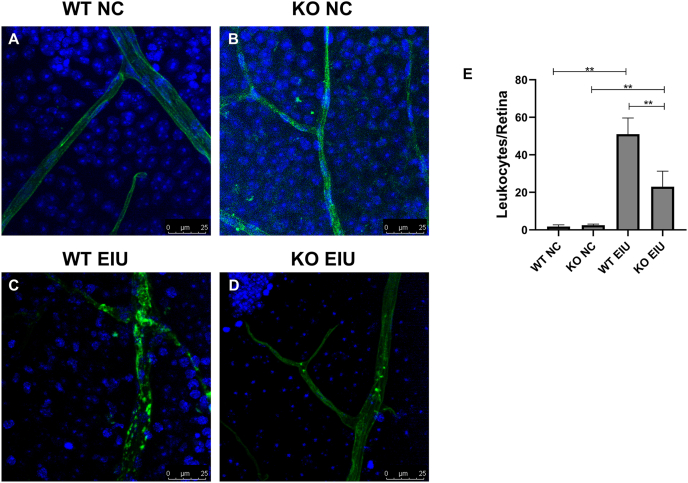
Knockout of cGAS reduced leukocyte adhesion in the retina. **(A)** Representative image of retinal leukostasis in the WT NC group. **(B)** Representative image of retinal leukostasis in the KO NC group. **(C)** Representative image of retinal leukostasis in the WT EIU group. **(D)** Representative image of retinal leukostasis in the KO EIU group. **(E)** Quantification of adherent leukocytes in the retinal vasculature. ∗*P* < 0.05, ∗∗*P* < 0.01. *n* = 6 biological replicates per group. WT, wild type; KO, Cgas knockout; NC, normal control; EIU, endotoxin-induced uveitis.

The adherence of leukocytes to the retinal vasculature contributes to the development of retinal inflammatory diseases such as diabetic retinopathy and ischemia-induced retinopathy. Therefore, we evaluated leukocyte recruitment and adhesion to retinal vessels in these groups. After concanavalin A perfusion, the adherence of leukocytes to retinal vessels was observed in retinal flat mounts from the WT EIU group but not in most flat mounts from the WT NC and KO NC groups (Fig. 5A, B). However, significant retinal leukostasis was observed in the WT EIU group (Fig. 5C). In contrast, the retinas of the KO EIU group (Fig. 5D) presented a significant decrease in retinal leukostasis compared with those of the WT EIU group (WT EIU *vs*. KO EIU: 51.00 ± 8.60 *vs*. 23.00 ± 8.29, *P* = 0.008; Fig. 5E).

**Figure 6:**
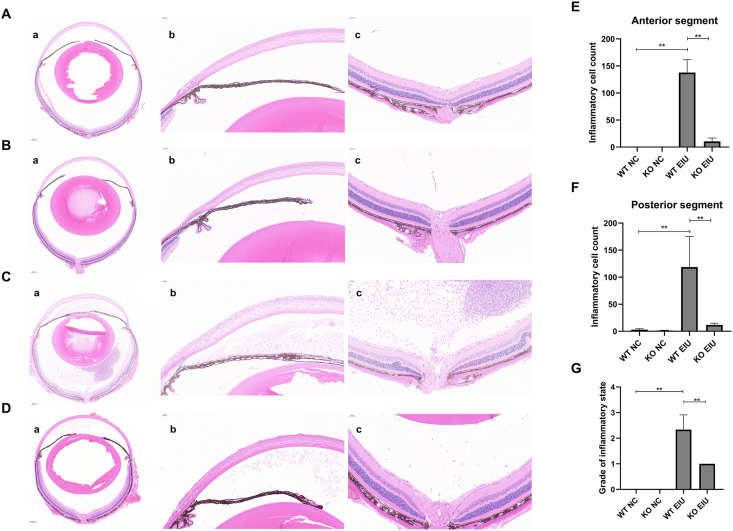
Hematoxylin and eosin staining and histological evaluation of the anterior and posterior segments. **(A)** No signs of infiltration in either segment in the WT NC group. **(B)** No signs of infiltration in either segment in the KO NC group. **(C)** Severe inflammatory cellular infiltration was visible in the anterior and posterior segments in the WT EIU group. **(D)** Inflammatory cellular infiltration was reduced in the anterior and posterior segments in the KO EIU group. (a–c) Histological sections of the total eyeball (a), iris-ciliary body (b), and retina (c) tissues. **(E)** Inflammatory cell count in the anterior segment. **(F)** Inflammatory cell count in the posterior segment. **(G)** Histologic grading. ∗*P* < 0.05, ∗∗*P* < 0.01. *n* = 3 biological replicates per group. WT, wild type; KO, Cgas knockout; NC, normal control; EIU, endotoxin-induced uveitis.

For histopathological evaluation, retinal tissues from each group were stained with hematoxylin and eosin and graded as described earlier (Fig. 6). The morphological data revealed no cellular infiltration in either the anterior or posterior segments of the WT NC and KO NC groups (Fig. 6A, B). Cgas KO significantly reduced inflammatory cell infiltration in the anterior segment (WT EIU *vs*. KO EIU: 137.67 ± 23.86 *vs*. 10.33 ± 6.43, *P* < 0.0001) and posterior segment (WT EIU *vs*. KO EIU: 118.67 ± 56.61 *vs*. 11.67 ± 3.21, *P* < 0.0074) in the EIU model (Fig. 6C–F). The mean histopathological score in the WT EIU group was 2.33 ± 0.58, whereas it was significantly lower (*P* = 0.0021) than that in the KO EIU group (Fig. 6G).

### Cgas KO decreased the activation of retinal macrophages and microglia

**Figure 7:**
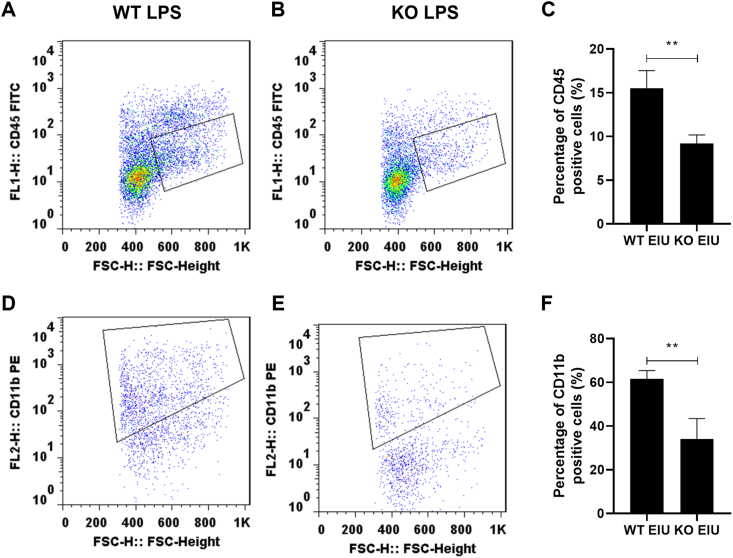
Retinal macrophages in each group were subjected to intravitreal injection for 48 h. **(A, B)** Sequential gating of CD45 med populations in WT EIU (A) and KO EIU (B). **(C)** Quantification of CD45 med cells. **(D, E)** CD11b^+^ analysis within the CD45 med gate in WT EIU (D) and KO EIU (E). **(F)** Percentage of CD11b^+^ cells. Student’s *t*-test, *n* = 3, ∗∗*P* < 0.01. WT, wild type; KO, Cgas knockout; NC, normal control; EIU, endotoxin-induced uveitis.

**Figure 8:**
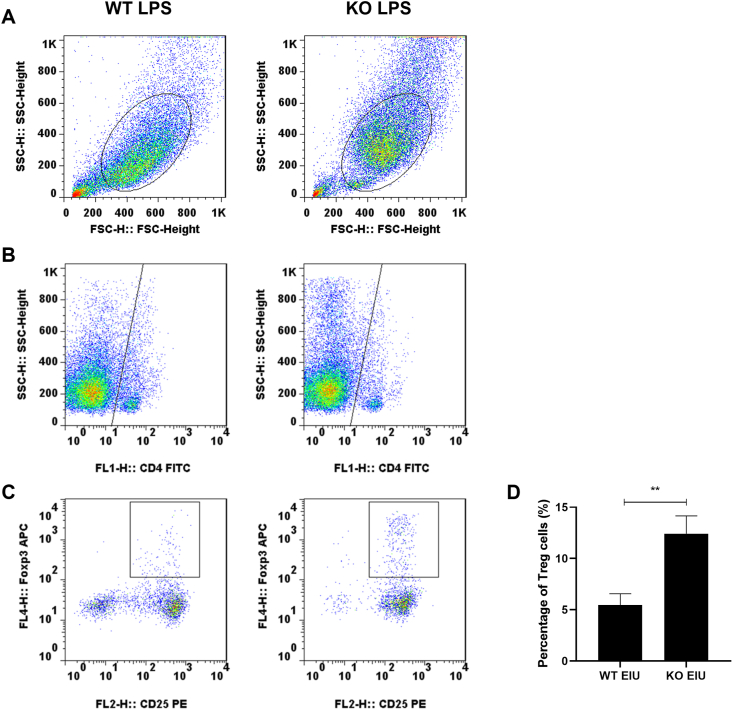
Tregs in each group were subjected to intravitreal injection for 48 h. **(A)** Gating on Foxp3+ cells. **(B)** Quantification of CD4 med cells. **(C)** Quantification of CD25 med cells. **(D)** Percentage of Treg cells. Student’s t-test, *n* = 3, ∗∗*P* < 0.01. WT, wild type; KO, Cgas knockout; NC, normal control; EIU, endotoxin-induced uveitis.

To further investigate how cGAS KO impacted the recruitment and activation of retinal macrophages in the EIU model, we assessed changes in CD45^+^ and CD11b^+^ populations. As shown in Figure 7, WT EIU mice presented significantly greater percentages of CD45 med cells (WT EIU *vs*. KO EIU: 15.53% ± 1.16% *vs*. 9.20% ± 0.56%, *P* < 0.01) and CD11b^+^ cells (WT EIU *vs*. KO EIU: 61.66% ± 2.15% *vs*. 34.11% ± 5.39%, *P* < 0.01) than did KO EIU mice. These findings suggest that cGAS KO may attenuate the inflammatory response, in part by reducing the shift in macrophages and the activation of retinal microglia. To provide a more complete understanding of immune modulation by cGAS deficiency, we performed additional flow cytometry analyses to specifically examine CD4^+^ CD25^+^ Foxp3^+^ Treg populations. As demonstrated in Figure 8, after gating on Foxp3^+^ cells, we identified Tregs via CD4^+^ and CD25^+^ markers. Our results revealed a significantly greater proportion of Tregs in the KO EIU group than in the WT EIU group (WT EIU *vs*. KO EIU: 5.46% ± 0.64% *vs*. 12.41% ± 1.00%, *P* < 0.01).

**Figure 9:**
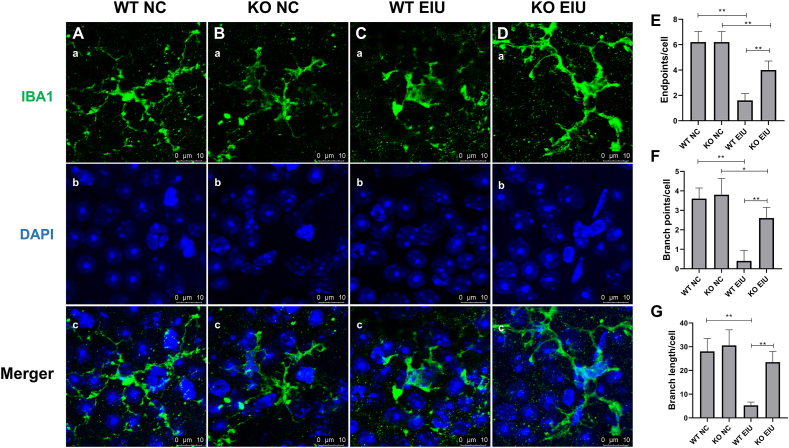
Immunofluorescence staining of microglial activation. **(A, B)** The morphology of microglia was more ramified in the WT NC (A) and KO NC (B) groups. **(C)** The cell bodies were enlarged in the WT EIU group. **(D)** The attenuation of microglial activation in the KO EIU group. (a–c) Fluorescence of IBA1 (green) (a), fluorescence of DAPI (blue) (b), and merged (c) images. **(E)** Endpoints/cell. **(F)** Branch points/cell. **(G)** Branch length/cell. *n* = 6, ∗*P* < 0.05, ∗∗*P* < 0.01. WT, wild type; KO, Cgas knockout; NC, normal control; EIU, endotoxin-induced uveitis.

As shown in Figure 9, immunofluorescence staining revealed that microglia in both WT NC and KO NC mice presented more endpoints and branch points and a more ramified morphology. In the WT EIU group (Fig. 9C), microglial cell bodies were enlarged, and their processes became shorter and thicker, characteristic of activated microglia. In contrast, microglia in the KO EIU group presented increased endpoints per cell, branch points per cell, and branch length per cell compared with those in the WT EIU group, suggesting a reduction in microglial activation (Fig. 9E–G).

### Differentially expressed mRNAs and GO enrichment analysis in the KO EIU and WT EIU groups

**Figure 10:**
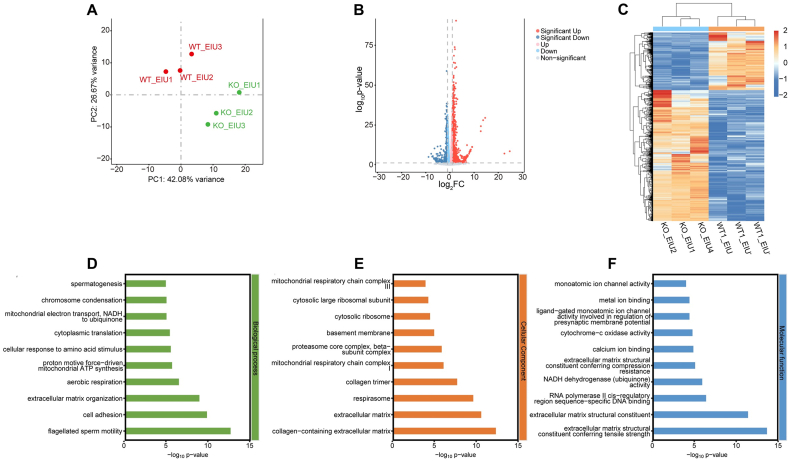
Analysis and confirmation of the differentially expressed mRNAs in the KO and WT EIU groups. **(A)** Principal component analysis of retinal mRNA expression levels in the KO EIU and WT EIU groups. The distribution of dots indicates distinct mRNA expression patterns between KO EIU and WT EIU. **(B)** Volcano plot of total mRNAs. The red, blue, and gray dots represent up-regulated, down-regulated, and non-significantly altered mRNAs, respectively. The data at infinity are not shown. **(C)** Hierarchical clustering heatmap analysis of differentially expressed mRNAs. The red represents increased expression, and the blue represents decreased expression. **(D**–**F)** GO enrichment analysis of co-expressed mRNA targets, covering biological processes, cellular components, and molecular functions. The top 10 GO terms of each domain are shown. WT, wild type; KO, Cgas knockout; NC, normal control; EIU, endotoxin-induced uveitis; GO, gene ontology.

To further investigate the effects of cGAS KO in the EIU model, bulk RNA sequencing was performed on KO and WT mice in the EIU model. Principal component analysis revealed a clear separation between the KO EIU and WT EIU groups (Fig. 10A). Bulk RNA sequencing revealed 2034 differentially expressed mRNAs, with 621 down-regulated and 1413 up-regulated (Table S5). The volcano plot and hierarchical clustering heatmap of these mRNAs are shown in Figure 10B and C.

GO enrichment analysis revealed 539 significantly enriched GO terms in the KO EIU group compared with the WT EIU group (Table S6). The top ten enriched GO terms across biological processes, molecular functions, and cellular components are presented in Figure 10D–F. Notably, the KO EIU group was significantly enriched in GO terms related to immune cell functions, including neutrophil chemotaxis (GO:0030593, *P* < 0.01), granulocyte differentiation (GO:0030851, *P* < 0.01), lymphocyte differentiation (GO:0030098, *P* < 0.01), lymphocyte proliferation (GO:0046651, *P* < 0.01), leukocyte chemotaxis (GO:0030595, *P* < 0.01), T-cell proliferation (GO:0042098, *P* = 0.03), cell adhesion (GO:0007155, *P* < 0.01), and cell migration (GO:0016477, *P* < 0.01). These findings suggest that cGAS KO may affect the function of immune cells in the retina, thereby modulating retinal inflammation.

Additionally, many genes involved in cellular energy metabolism were significantly enriched in the KO EIU group, including terms related to the respirasome (GO:0070469, *P* < 0.01), aerobic respiration (GO:0009060, *P* < 0.01), mitochondrial respiratory chain complex I (GO:0005747, *P* < 0.01), proton motive force-driven mitochondrial ATP synthesis (GO:0042776, *P* < 0.01), ATP synthesis coupled electron transport (GO:0042773, *P* < 0.01), mitochondrial respiratory chain complex III (GO:0005750, *P* < 0.01), electron transport coupled proton transport (GO:0015990, *P* < 0.01), mitochondrial respiratory chain complex I assembly (GO:0032981, *P* < 0.01), and mitochondrial respiratory chain complex IV (GO:0005751, *P* < 0.01). These enriched genes suggest that cGAS KO may also modulate retinal inflammation through alterations in the energy metabolism of retinal cells. However, further studies are needed to validate this hypothesis.

## Discussion

This study examined the role of the cGAS‒STING pathway in retinal inflammation via bulk RNA sequencing. We observed mtDNA release and activation of the downstream TBK1‒IRF3 signaling pathway in the EIU model. Additionally, Cgas KO significantly diminished the ocular inflammatory response triggered by intravitreal LPS injection.

The cGAS-STING pathway has become a pivotal mechanism that links DNA sensing to the activation of robust innate immune responses.^28^, ^29^, ^30^ In this pathway, the binding of cGAS to double-stranded DNA triggers its catalytic activity, leading to the generation of cGAMP. This molecule then functions as a strong activator of STING.^30^^,^^31^ STING activation leads to the phosphorylation of nuclear transcription factors and the up-regulation of type I interferons, thereby amplifying inflammatory signals and triggering the cellular inflammatory response.^1^ Consequently, the cGAS-STING signaling pathway is involved in a variety of diseases, including infections, autoimmune disorders, cancers, fibrosis, and neurodegenerative conditions. Our GO enrichment analysis of expressed mRNA targets revealed significant enrichment of genes related to cellular responses to exogenous dsDNA and type I interferon production in the EIU retina compared with the normal retina. These findings indicate that the cGAS-STING pathway might mediate retinal injury in the EIU model by linking exogenous dsDNA to type I interferon production.

The mechanism underlying cGAS-STING pathway activation in EIU retinal injury was also explored. Previous research has shown that pathological conditions lead to the escape of mtDNA into the cytosol, where it is detected by cGAS as exogenous dsDNA, thereby activating the cGAS-STING pathway.^17^ Current studies identify cGAS as a cytosolic nucleotide-sensing molecule. Our prior work demonstrated that pathological stimuli induce mitochondrial Ca^2+^ influx and morphological alterations, triggering opening of the mitochondrial permeability transition pore.^32^ This process releases cytochrome C and mtDNA into the cytosol via the collapse of the membrane potential.^33^ Structurally, cGAS contains a nucleotidyltransferase domain and two DNA-binding domains that recognize dsDNA, catalyzing cGAMP synthesis from ATP/GTP.^1^ As a critical cytosolic DNA sensor, cGAS initiates the cGAMP-STING signaling cascade through cGAMP-mediated second messenger activity, followed by p62-dependent ubiquitin‒proteasome degradation.^34^

Real-time PCR revealed a notable increase in cytosolic mtDNA in the EIU retina. Additionally, GSEA revealed significant enrichment of gene sets related to the cytosolic DNA-sensing pathway in the EIU retina. Thus, we hypothesize that LPS injection into the vitreous body leads to mtDNA release into the cytoplasm, subsequently activating the cytosolic DNA-sensing pathway via the cGAS‒STING pathway, which then amplifies inflammatory signals, resulting in retinal inflammation and damage. Western blotting analysis further supported the activation of the cGAS‒STING‒TBK1‒IRF3 signaling pathway in the EIU model. Thus, our findings indicate that LPS-induced mtDNA release activates the cGAS‒STING‒TBK1‒IRF3 pathway, contributing to retinal inflammation and injury.

To explore the role of the cGAS-STING pathway in endotoxin-induced uveitis, we employed Cgas KO mice and analyzed changes in inflammatory responses. Cgas KO significantly alleviated ocular inflammation, including improved ocular inflammation, reduced retinal vascular leakage, decreased leukocyte adhesion, and inhibited infiltration and activation of macrophages in the retina. Additionally, the activation of retinal microglia was significantly improved. Recent studies have shown that chronic inflammation induced by the cGAS‒STING pathway drives the development of various diseases,^30^ such as systemic lupus erythematosus,^35^ rheumatoid arthritis,^36^ atherosclerosis,^37^ myocardial infarction,^38^ diabetes,^39^ obesity,^40^ Alzheimer’s disease,^41^ Parkinson’s disease,^10^ liver cirrhosis,^42^ and sepsis.^43^ In our previous studies, we have demonstrated *in vitro* that the cGAS‒STING pathway contributes to inflammatory damage in retinal vascular endothelial cells.^17^ Our current *in vivo* research using animal models confirmed that the cGAS‒STING pathway is involved in retinal inflammatory damage, suggesting that targeting this pathway could be a promising approach for treating uveitis.

Our prior work has demonstrated that mtDNA activates the cGAS-STING pathway, inducing nuclear factor kappa B (NF-κB) phosphorylation and nuclear translocation.^17^ This cascade drives proinflammatory gene expression through TBK1-mediated NF-κB/IRF3 activation, which concurrently up-regulates cytokines (CCL4, CXCL10) and type I interferons (IFN-β1).^44^ Phosphorylated IRF3 initiates signal transducer and activator of transcription 6 (STAT6) activation, amplifying IFN-β1 production, a process also observed in Toll-like receptor 4 (TLR4)-mediated blood–retinal barrier breakdown during diabetic retinopathy.^45^^,^^46^ Activated NF-κB directly binds to the IRF1 promoter, stimulating pathogen defense genes while synergizing with NOD-, LRR- and pyrin domain-containing protein 3 (NLRP3) inflammasome activation. Notably, LPS-induced mtDNA replication mirrors our isolated mtDNA treatment results, confirming conserved innate immune activation via Toll-like receptors on retinal pigment epithelial cells.^47^

To further explore the mechanisms by which Cgas KO alleviates ocular inflammation, bulk RNA sequencing was performed on KO and WT EIU model mice. Cgas KO significantly enriched genes related to immune cell functions, including neutrophil chemotaxis, granulocyte differentiation, lymphocyte differentiation, lymphocyte proliferation, leukocyte chemotaxis, T-cell proliferation, cell adhesion, and cell migration, according to the GO terms. These findings indicate that Cgas KO may regulate retinal inflammation by affecting the functions of immune cells in the retina. Moreover, many genes associated with cellular energy metabolism, including those related to the respirasome, were significantly enriched by Cgas KO. These findings suggest that Cgas KO may also modulate retinal inflammation through changes in the energy metabolism of retinal cells. However, further studies are needed to validate this hypothesis.

Although the canonical cGAS‒STING pathway is a well-established inflammatory signaling cascade, and we hypothesize that its activation occurs in the EIU model, our study lacks direct evidence from STING KO mice. Thus, we cannot definitively conclude that STING is the exclusive downstream effector of cGAS in EIU. Future experiments with STING KO mice will help determine the dependency on STING and rule out potential off-target or STING-independent effects of cGAS.

In summary, our study revealed a marked decrease in EIU severity in Cgas KO mice, highlighting the critical role of the cGAS-STING pathway in this retinal inflammation model. However, the exact mechanisms by which cGAS-STING influences retinal inflammatory diseases, particularly the upstream pathways, remain unclear and warrant further research. We are currently conducting bulk RNA sequencing on KO EIU and WT EIU models to gain deeper insight into the role of the cGAS-STING pathway in retinal inflammation. Future studies should prioritize the development of cGAS-targeted gene therapies for uveitis patients refractory to current pharmacological regimens, particularly to address unmet needs in treatment-resistant populations.

In conclusion, our findings provide evidence that the cGAS‒STING pathway is activated in a model of LPS-induced ocular inflammation and that cGAS KO effectively inhibits this inflammation. These results suggest that the cGAS-STING pathway is crucial in ocular inflammatory responses and could represent a novel target for therapeutic intervention in ocular inflammatory diseases.

## CRediT authorship contribution statement

**Yue Guo:** Methodology, Data curation, Writing – original draft, Formal analysis. **Ruiping Gu:** Visualization, Writing – original draft, Supervision, Data curation, Formal analysis. **Jiaojiao Wei:** Supervision, Formal analysis, Writing – review & editing, Methodology. **Chunhui Jiang:** Supervision, Writing – review & editing, Methodology, Funding acquisition, Investigation.

## Data availability

The original contributions presented in this study are included in the article/supplementary material; further inquiries can be directed to the corresponding author.

## Ethics declaration

This study was approved by the Ethics Committee of the Eye and Ear Nose Throat Hospital of Fudan University, Shanghai, China.

## Funding

This study was supported by the 10.13039/501100012166National Key R&D Program of China (No. 2022YFC2404203), the 10.13039/501100001809National Natural Science Foundation of China (No. 82070980), and the Shanghai Hospital Development Center Foundation (China) (No. SHDC12023116).

## Conflict of interests

The authors declared no conflict of interests.
