# Supplementary material for: cGAS knockout inhibited endotoxin-induced uveitis in mice
Source: Genes Dis. 2025 Jul 29;13(2):101786. doi: 10.1016/j.gendis.2025.101786 (PMC12757528; doi:10.1016/j.gendis.2025.101786)
Supplement: Multimedia component 1 [file mmc1.docx]

Supplement table 1: Sequence primer

| Gene |  |  |
| --- | --- | --- |
| 18S rDNA | Forward | 5′-AGTTATGGTTCCTTTGGTCGCT-3′ |
|  | Reverse | 5′-TCTGATAAATGCACGCATCCC-3′ |
| mt-Co1 | Forward | 5′-CACTATTATCAACATGAAACCCCC-3′ |
|  | Reverse | 5′-AGAATATAAACTTCTGGGTGCCC-3′ |

| Supplement table 2: Antibodies used in the Study |  | |  |
| --- | --- | --- | --- |
| Antibody | Source | Identifier |  |
| anti-cGAS antibody (1:1000) | Abcam, Cambridge, MA, USA | Cat# ab179785 |  |
| anti-STING (D1V5L) Rabbit mAb (1:1000) | Cell Signaling Technology, Boston, USA | Cat# 50494 |  |
| anti-TBK1/NAK (D1B4) rabbit mAb (1:1000) | Cell Signaling Technology, Boston, USA | Cat# 3504 |  |
| anti-phospho-TBK1/NAK (Ser172) (D52C2) XP® rabbit mAb (1:1000) | Cell Signaling Technology, Boston, USA | Cat# 5483 |  |
| anti-IRF3 (D83B9) rabbit mAb (1:1000) | Cell Signaling Technology, Boston, USA | Cat# 4302 |  |
| anti-phospho-IRF3 (Ser396) (D6O1M) rabbit mAb (1:1000) | Cell Signaling Technology, Boston, USA | Cat# 29047 |  |
| horseradish-peroxidase-conjugated goat anti-rabbit IgG H&L (1:5000) | Abcam, Cambridge, MA, USA | Cat# ab205718 |  |
